# Proteogenomic analysis of Cyprinid herpesvirus 2 using high-resolution mass spectrometry

**DOI:** 10.1128/jvi.01960-24

**Published:** 2025-04-02

**Authors:** Chen Xu, Fangxing Yu, Mingyang Xue, Zhenyu Huang, Nan Jiang, Yiqun Li, Yan Meng, Wenzhi Liu, Ya Zheng, Yuding Fan, Yong Zhou

**Affiliations:** 1 Yangtze River Fisheries Research Institute, Chinese Academy of Fishery Sciences 499154 , Wuhan, China; 2 College of Life Sciences, Shanghai Normal University 124445 , Shanghai, China; University of Virginia, Charlottesville, Virginia, USA

**Keywords:** Cyprinid herpesvirus 2, proteogenomics, gene discovery, mass spectrometry

## Abstract

Cyprinid herpesvirus 2 (CyHV-2) is the main pathogen responsible for the development of herpesviral hematopoietic necrosis disease (HVHND) in crucian carp (*Carassius auratus*). The CyHV-2 genome encodes approximately 150 genes that are expressed in a well-defined manner during productive infection. However, CyHV-2 open reading frames (ORFs) are primarily derived from sequence and homology analyses, and most lack protein-level evidence to support their properties. In this study, we used high-resolution mass spectrometry followed by proteogenomic mapping to achieve genome re-annotation of CyHV-2. Based on our results, a total of 1,683 MS/MS spectra could be mapped to the CyHV-2 genome through six-frame translation, with 1,665 corresponding to 117 currently annotated protein-coding ORFs. Three of the remaining 18 peptides were mapped to the N-terminal extension region of known ORFs. However, 12 novel CyHV-2 ORFs, designated nORF1-12, were identified and characterized for the first time based on the remaining 15 peptides that could be mapped to previously unannotated regions of the viral genome. And the sequence differences of the novel phosphorylated nORF1, also referred to as ORF25E, in different CyHV-2 strains indicated that the nORF1 is a prospective molecular marker that can monitor the evolution from the Japan (J) to the China (C) genotype of CyHV-2. These findings further validate existing annotations, expand the genomic landscape of CyHV-2, and provide a rich resource for aquatic virology research.

**IMPORTANCE:**

CyHV-2 is a viral pathogen that poses a significant threat to crucian carp farming. CyHV-2 has a large genome with complex sequence features and diverse coding mechanisms, which complicates accurate genome annotation in the absence of protein-level evidence. Here, we employed various protein extraction and separation methods to increase viral protein coverage and performed an integrated proteogenomic analysis to refine the CyHV-2 genome annotation. A total of 129 viral genes were confidently identified, including 117 currently annotated genes and 12 novel genes. For the first time, we present large-scale evidence of peptide presence and levels in the genome of aquatic viruses and confirm the majority of the predicted proteins in CyHV-2. Our findings enhance the understanding of the CyHV-2 genome structure and provide valuable insights for future studies on CyHV-2 biology.

## INTRODUCTION

Cyprinid herpesviruses comprise a class of linear, double-stranded DNA viruses that are members of the genus *Cyprinivirus*, family *Alloherpesviridae*, including carp pox cyprinid herpesvirus 1 (CyHV-1), cyprinid herpesvirus 2 (CyHV-2), and koi herpesvirus cyprinid herpesvirus 3 (CyHV-3) (1). CyHV-2 specifically infects crucian carp or goldfish (*Carassius auratus*), causing severe hemorrhages at various body sites, such as gills, eyes, fins, and swollen anus, a condition known as herpesviral hematopoietic necrosis disease (HVHND) (2). This disease was first documented in juvenile goldfish in Japan in 1992 and 1993 (3). Since then, cases of CyHV-2 infections have been reported in various countries worldwide, including Hungary, the Czech Republic, Italy, Switzerland, Germany, Turkey, and Thailand, resulting in substantial economic losses in the aquaculture industry (4–6). In China, CyHV-2 was first detected in ornamental goldfish in 2012, and since then, HVHND has been reported almost every year across the country (7).

The first complete genome of CyHV-2 (ST-J1 strain) was sequenced in 2013. It comprises 290,304 bp, encoding 150 putative open reading frames (ORFs), and a unique region flanked at each terminus by a sizable direct repeat (1). Until now, seven CyHV-2 strain genomes have been fully sequenced (namely, STJ1, SY-C1, CaHV, SY, YZ-01, CNDF-TB2015, and YC-01) with genomic sequence analyses revealing that all CyHV-2 strains share more than 98% homology (1, 7–10). Comparative genomics analyses revealed numerous mutations, including insertions, deletions, and rearrangements that are widely present in the different CyHV-2 strains, resulting in CyHV-2 strains being divided into China (C) and Japan (J) genotypes (7). The ST-J1 strain is classified as a J genotype, whereas the remaining strains are classified as C genotypes. For example, variations in *ORF10*, *ORF25B*, *TK* (*ORF55*), *ORF63*, *ORF71*, *ORF79*, *ORF107*, and *ORF156* in different CyHV-2 strains are considered potential molecular genetic markers to distinguish C and J genotypes, as well as different Chinese CyHV-2 isolates (7,8,10). Except for the aforementioned variable proteins, twelve core CyHV-2 ORFs, involved in DNA replication, DNA packaging, and capsid morphogenesis, are highly conserved in all sequenced Alloherpesviridae members and were presumably inherited from a common ancestor (11). All 150 predicted ORFs in the CyHV-2 genome are expressed sequentially based on the transcription data and can be divided into three temporal phases: immediate–early, early, and late genes (12). However, only 74 CyHV-2 ORFs, comprising three capsid proteins, 18 membrane proteins, and 53 other proteins, are regarded as viral structural proteins based on their detection by mass spectrometry from purified particles of CyHV-2 (13).

The functional protein-coding ORFs in the original CyHV-2 sequence (ST-J1 strain) were predicted using a range of standard bioinformatic tools in the genome annotation process of anguillid herpesvirus 1 (AngHV1) and cyprinid herpesvirus 3 (CyHV-3) (1, 14). Consequently, certain annotated ORFs may not encode functional proteins, and there exists a possibility that some functional protein-coding ORFs have been omitted. This particularly encompasses short ORFs (<100 amino acids), those overlapping with other ORFs, or those utilizing noncanonical initiation codons (non-ATG). Evidence of protein-level gene expression is ultimately required to validate the presence of a predicted protein-coding gene (15). Thus far, the CyHV-2 genome annotation’s accuracy regarding its predicted ORFs and the actual encoded protein remains inadequate.

Proteogenomic mapping has been regarded as a promising strategy to improve genome annotation by providing experimental evidence at a proteomic level (16). By matching identified peptides to the corresponding genome, gene predictions can be improved, single-amino acid variants can be identified, and novel protein-coding genes can be uncovered. Since “proteogenomics” was first proposed as an approach in the literature in 2004 (17), it has been successfully implemented to enhance genome annotation in many organisms, including prokaryotes, eukaryotes, and viruses (18–21). Proteogenomics research is continuously advancing due to improvements in deep MS-based proteomic and computational techniques (16). However, challenges remain in the current popular bottom–up proteomics strategy, especially regarding the detection of low-molecular weight proteins, lowly expressed proteins, and those that possess transmembrane structures. Necessary steps are needed to achieve better protein identification results in bottom–up proteomic approaches, such as refining protein extraction, reducing sample complexity, and incorporating multiple search engine information for annotation.

In this study, we utilized multiple protein extraction and separation methods to increase viral protein coverage. We then mapped the identified peptides to the CyHV-2 six-frame translated proteome, aiming to improve CyHV-2 genome annotation. All peptides that were mapped to predicted existing ORFs or previously unannotated viral genome regions were comprehensively covered. Our findings would contribute to a comprehensive understanding of CyHV-2 genome structure and provide novel insights for further studying CyHV-2 biology.

## MATERIALS AND METHODS

### Protein extraction

Healthy crucian carps (mean weight, 150 ± 50 g) were intraperitoneally injected with 200 µL volumes (1 × 10^6^ copies/µL) of the CyHV-2 YC2022 strain, which was isolated from diseased gibel carps in Sheyang county, China, in 2022 (22). The presence of CyHV-2 in the fish was assessed by PCR in accordance with the Chinese quarantine methods of epidemic diseases in fish at 5 days post-infection. Two different lysis buffers were used for protein extraction from the kidneys following three washes with PBS to remove the blood: (i) 50 mM HCl, 0.5% dithiothreitol (DTT), and 0.1% N-dodecyl-β-D-maltoside (DDM); (ii) 20 mM Tris-HCl (pH = 7.5), 150 mM NaCl, 1 mM EDTA, 0.1% DDM, and 1 × Roche protease & phosphatase inhibitor cocktail (Roche, Switzerland). The samples were homogenized, sonicated for 5 minutes on ice, and centrifuged at 12,000 *g* at 4°C for 10 minutes to remove the cell debris or precipitated proteins resulting from acid denaturation. The supernatant extracted using the first method was further filtered using a 10 kDa ultrafiltration device to remove high MW proteins and then evaporated to dryness at low temperature. The supernatant extracted using the second method was precipitated overnight at −20°C using five volumes of precooled acetone and then washed with an equivalent volume of precooled methanol. The protein concentration was determined using the BCA protein assay kit (Beyotime, China).

### Trypsin digestion

The protein pellets obtained using the two lysis buffers were dissolved in 6 M urea and reduced in 10 mM DTT at 37°C for 40 minutes. The pellets were then alkylated with 15 mM iodoacetamide at 37°C in the dark for 30 minutes. After diluting the samples with 50 mM NH_4_HCO_3_ to a final urea concentration of 1 M, trypsin (Promega) was added at a 1:50 (wt/wt) ratio and incubated for 12 hours at 37°C for protein digestion.

### High pH reverse-phase separation

All peptides were fractionated using C18 columns (Agilent Technologies) as previously described (23). In brief, the tryptic peptides were dissolved in 25 mM NH_4_FA (pH = 10), loaded onto the C18 columns, equilibrated with 25 mM NH_4_FA (pH = 10), and then eluted with a series of elution buffers containing different concentrations of ACN (5%, 10%, 20%, 30%, 40%, and 75%) in 25 mM NH_4_FA (pH = 10). All fractions were freeze-dried and resuspended in 0.1% formic acid for LC-MS/MS analysis.

### Mass spectrometry data acquisition

All peptides were analyzed by a Q-Exactive Plus mass spectrometer equipped with an EASY-nLC 1200 system. Peptides were separated using a C18 nanotrap column (Acclaim PepMap C18; 75 µm × 25 cm, 2 µm, 100 Å, Thermo Scientific) at a flow rate of 300 nL/minute through a 120 min linear gradient from 5% solvent B (100% ACN/0.1% formic acid, vol/vol) to 80% solvent B. Peptides were ionized at 2.2 kV using nanoelectrospray ionization. Data-dependent acquisition (DDA) was performed by acquiring a full scan range of 350–2,000 m/z in the Orbitrap at a resolving power of 70,000 (@200 m/z). The MS/MS scans were analyzed using an isolation width of 1.8 m/z, with an AGC target of 5 × 10^4^ at a resolving power of 17,500, a 50 ms maximum ion injection period, 28% normalized HCD collision energy, and 20 MS/MS scans per cycle. The dynamic exclusion period was set at 40 seconds.

### Sequence database development and peptide identification

Proteome Discoverer 2.4 and pFind 3.2 (24) were employed to analyze all RAW data files and to search a customized protein database containing the crucian carp’s proteome and the full CyHV-2 YZ-01 strain genome sequence translated in all six reading frames, as generated by the ORFfinder software (https://www.ncbi.nlm.nih.gov/orffinder/). A total of 5,914 identified predicted ORFs are listed in Table S1. The criteria for the retrieved data were set as follows: (i) the MS1 mass tolerance and MS2 mass tolerance in Proteome Discoverer 2.4 were 10 ppm and 0.02 Da, respectively; (ii) the false discovery rate was 1%; (iii) trypsin was the cleavage enzyme allowing up to two missed cleavages; (iv) the carbamidomethyl modification of cysteines was set as a fixed modification; and (v) the variable modifications included methionine oxidation, protein N-terminal acetylation, and serine, threonine, and tyrosine phosphorylation modifications.

All identified peptides from two different search engines were first manually merged and mapped to the predicted CyHV-2 protein database and the crucian carp’s proteome using the Peptide search tool in UniProt (https://www.uniprot.org/peptide-search). The genomic mapping information of peptides that fully matched the predicted CyHV-2 protein database and did not match the crucian carp’s proteome was recorded and used for subsequent analysis. The proteogenomic mapping results were visualized using TBtools software (25).

### Real-time PCR analysis

Total RNA was isolated from the kidneys of CyHV-2-infected fish using the FastPure Cell/Tissue Total RNA Isolation Kit (Vazyme, China) and was reverse-transcribed onto cDNA using the HiScript III First-Strand cDNA Synthesis Kit (Vazyme, China) according to the manufacturer’s instructions. Forward and reverse primers were designed to amplify the novel genes based on their coding regions. The primers are listed in Table S2.

### Plasmid construction, transfection, and confocal imaging

The full-length nORF1 sequence was amplified by PCR using viral cDNA library as templates and then cloned into plasmid pEGFP-N1 at *XhoI-PstI* sites. The resultant plasmid was extracted after sequencing validation using the endo-free plasmid Midi Kit (Omega, USA). Subsequently, the plasmid was transfected into a cell line from the gibel carp (GiCB) brain using Takara kits (Japan) as per the manufacturer’s instructions. The transfected GiCB cells were cultured in a Hyclone Medium 199 (Hyclone, USA) containing 10% fetal bovine serum (FBS) at 28°C. At 24 hours post-transfection, cells containing recombinant plasmids were first washed with PBS three times to remove the cell medium and then fixed with 4% paraformaldehyde for 20 minutes. Subsequently, the cell nuclei and cell membrane were stained using DAPI (1 µg/mL) and Dil (5 µM) reagents. Finally, the transfected cells were visualized using an Olympus confocal microscope (Japan).

## RESULTS

### Integrative proteogenomics workflow for annotating the CyHV-2 genome

**Fig 1:**
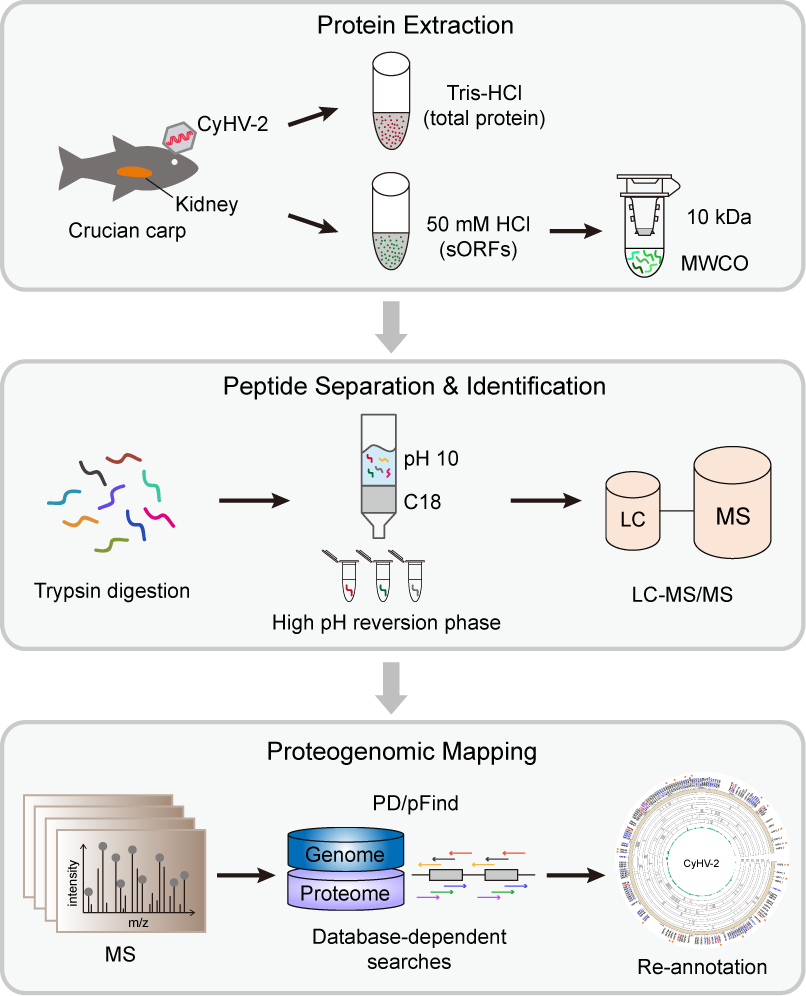
Workflow for the proteogenomic analysis of CyHV-2. Total proteins and sORFs were extracted from the kidneys of CyHV-2-infected crucian carp. All proteins were digested, fractionated, and subjected to LC-MS/MS. The mass spectra were searched against a customized fasta file containing the host’s and the viral six-frame translated proteome using two search engines to identify protein-coding genes.

LC-MS-/MS-based bottom–up proteomics was employed to examine the expressed proteome of CyHV-2. To promote viral protein identification and sequence coverage, we first utilized 50 mM HCl and Tris-HCl (pH = 7.5) buffers to improve the protein extraction efficiency, especially for sORFs (Fig. 1). The HCl buffer has been demonstrated to facilitate the precipitation of high-molecular weight (MW) proteins and enhance sORF enrichment (26–28). Then, the HCl lysate was filtered using a 10 kDa ultrafiltration device to further remove the high MW proteins. The total proteins and separated sORFs were digested with trypsin. Subsequently, the peptide mixtures from two groups were fractionated with high pH reverse-phase columns to reduce sample complexity and were prepared for MS analysis.

To maximize MS spectrum interpretation, we searched the MS database against our customized protein database using two different search engines, Proteome Discoverer and Open-pFind, applying a stringent filtering threshold (FDR ≤ 1%). The customized protein database was generated by combining crucian carp’s proteome and the CyHV-2 YZ-01 strain full genome sequence translated in all six reading frames. The CyHV-2 YZ-01 strain genome sequence was selected over that of other strains in the NCBI database as the isolated YC2022 strain in this study exhibited a higher sequence similarity with the YZ-01 strain, based on the phylogenetic tree constructed using the sequences of eight highly variable genes (Fig. S1; Table S3). After removing the peptides that can match the crucian carp’s genome, both host’s and viral genome, the remaining unique peptides were used to reannotate the CyHV-2 genome and discover potential novel ORFs.

### A draft map of the CyHV-2 proteome based on MS data

**Fig 2:**
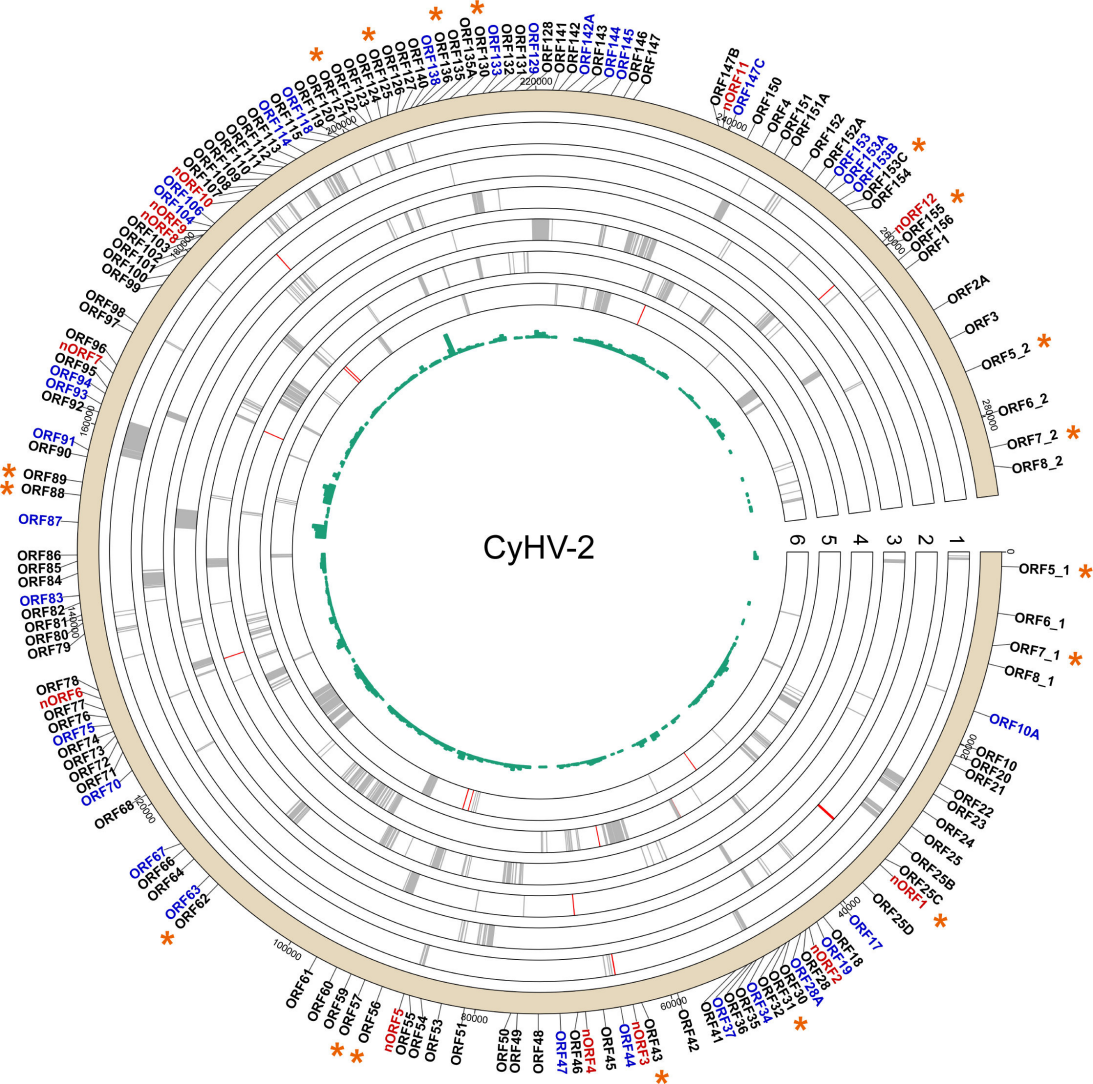
Overview of the CyHV-2 proteogenomic analysis results. The 288 kb CyHV-2 YZ-01 genome is displayed in the six possible reading frames; gene names are shown on the outside of the circle. All identified peptides are mapped to the CyHV-2 genome and shown with different colors. Gray: mapped to currently annotated protein-coding regions (referring to the annotation of both YZ-01 and ST-J1 strains); red: mapped to currently unannotated regions. The green histogram in the center represents the identified densities of genes that encode for peptides in different genome regions. A total of 27 currently annotated ORFs without peptide-level evidence are marked in blue. Twelve novel ORFs (nORFs) are marked in red. The asterisks indicate that the protein is phosphorylated.

Combining the retrieved results from Proteome Discoverer and Open-pFind yielded a total of 1,683 MS/MS spectra that completely matched peptides encoded by the CyHV-2 YZ-01 genome based on a six-frame translation, which were subsequently used for CyHV-2 genome re-annotation (Table S4). A total of 912 MS/MS spectra were identified with the HCl extraction method, while 1,328 MS/MS spectra were identified with the Tris extraction method. Among these, 557 MS/MS spectra were identified by both methods (Fig. S2). The MS/MS spectra that matched both the viral genome and the crucian carp’s genome were excluded from the analysis. Figure 2 illustrates the results of proteogenomic mapping, demonstrating that proteins are expressed in most regions of the CyHV-2 genome, with a significantly higher frequency of gene expression in the central region compared to both termini of the genome. Upon all identified MS/MS spectra, 1,665 were mapped to CyHV-2 currently annotated protein-coding ORFs (referring to the genome annotation of both ST-J1 and YZ-01 strains) corresponding to 117 known proteins. The remaining 18 MS/MS spectra were mapped to viral genomic regions with no current annotation, of which three were located in N-terminal extension regions of currently annotated proteins, nine were located in protein-coding regions based on a six-frame translation, and six were newly identified ORFs. Based on their genomic localization, the 18 newly identified peptides could be further classified as intergenic (seven peptides located between annotated gene models) or intragenic (11 peptides within or near an annotated gene model; Table 1). Excluding the three peptides located in N-terminal extension regions, the remaining 15 novel peptides constituted 12 potential novel ORFs, termed nORF1–12.

**TABLE 1:** List of identified novel peptides

Identified novel peptides	Position in CyHV-2 genome	Classification	Protein name	Initiation codon	Predicted domains
GDIGDSFDDRP	MK260012.1:34577-34545	Intergenic	nORF1	ATG	Disordered; region of a membrane-bound protein predicted to be embedded in the membrane
EPEEEEPQTFVEAF VASPLLLK	MK260012.1:34451-34386	Intergenic			
VASPLLLK	MK260012.1:34409-34386	Intergenic			
ESVPNWK	MK260012.1:44332-44312	intragenic	nORF2	CTG	/^*a*^
RTWLSAQQR	MK260012.1:63980-64006	Intergenic	nORF3	CTG	Disordered
KQAGITLK	MK260012.1:68976-68999	Intragenic	nORF4	TTG	Transmembrane region; cytoplasmic domain
VLKPDIK	MK260012.1:87073-87053	Intergenic	nORF5	CTG	Disordered
DIVISVPK	MK260012.1:87052-87029	Intergenic			
SEVLLLR	MK260012.1:131682-131662	Intragenic	nORF6	ATG	/
VLLVGSGTR	MK260012.1:166229-166255	Intragenic	nORF7	TTG	/
VQEELER	MK260012.1:182170-182150	Intragenic	nORF8	CTG	Signal peptide; non-cytoplasmic domain
LTTGHTLEYRPDPR	MK260012.1:182635-182594	Intragenic	nORF9	TTG	Signal peptide; non-cytoplasmic domain
VAADVGALGSVGR	MK260012.1:186581-186543	Intragenic	nORF10	CTG	/
ELSQFTAMVR	MK260012.1:239347-239318	Intragenic	nORF11	ATG	/
MILALVQAK	MK260012.1:259457-259431	Intragenic	nORF12	ATG	Disordered
SQPPMPECIFQK	MK260012.1:50588-50623	Intragenic	ORF36	CTG	Disordered
SSNDDGGPPVPFIER	MK260012.1:65158-65202	Intragenic	ORF45	CTG	Disordered
IQTPFK	MK260012.1:86071-86054	Intergenic	ORF54	TTG	Disordered

^*a*^ /, no predicted domain.

Based on bioinformatic analyses, the CyHV-2 YZ-01 genome has been predicted to encode 148 genes. A total of 121 gene-encoded proteins have been identified based on the MS results in this study and previous structural protein studies, while only four structural proteins (ORF19, ORF44, ORF70, and ORF83) were not identified in our data set (13). Meanwhile, 27 predicted ORFs still lack protein-level evidence to support them (Fig. 2). Besides, we observed that the currently known ORFs in CyHV-2 are predominantly derived from canonical (ATG) initiation codon annotations, potentially omitting ORFs with noncanonical (non-ATG) initiation codons. Interestingly, the newly identified peptides corresponding to the eight nORFs and the three ORFs with revised annotations possessed non-ATG initiation codons such as TTG and CTG (Table 1). These results enhance our understanding of the proteins encoded by the viral genome.

### Validation of novel peptides with surrounding viable ORFs

**Fig 3:**
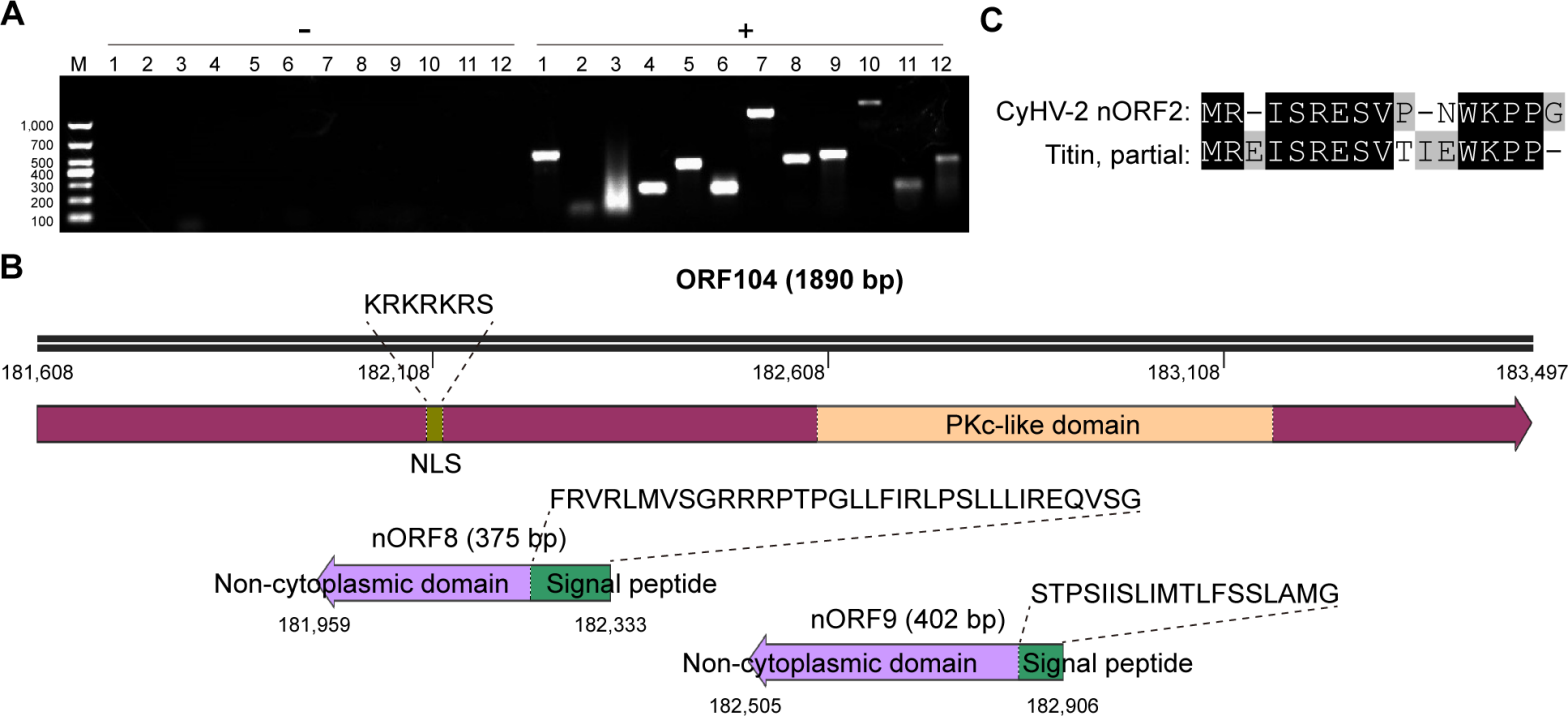
Sequence analysis of the identified novel peptides in the CyHV-2 proteome. (**A**) Validation of 12 nORFs using RT-PCR. Total mRNA subjected (+) or not (–) to reverse transcription served as a template. (**B**) Functional analysis of ORF104, nORF8, and nORF9 in the CyHV-2 YZ-01 strain. The online InterPro tool was used to predict protein domains and other functionally important sites. (**C**) Sequence alignment between CyHV-2 nORF2 and the insect protein titin from species including *Pieris brassicae*, *Amyelois transitella*, *Vanessa atalanta*, *Leguminivora glycinivorella*, and *Pieris rapae*. The sequence fragment depicted is highly conserved across all of the above insect species.

To validate the annotation accuracy of the 12 nORFs, we manually checked that almost all the novel peptides, except one, uniquely matched to corresponding protein-coding regions, and all were supported by rich b/y ion series. The RNA transcripts of 12 nORFs were detected by RT-PCR. Most of them were expressed during viral proliferation, apart from nORF2 and nORF3 (Fig. 3A). Total mRNA that was not reverse-transcribed was used directly as a template for PCR amplification, serving as a negative control to exclude potential genomic DNA contamination (Fig. 3A). It is generally assumed that the occurrence of conserved domains or homologs provides further evidence for the existence of novel protein-coding regions. Therefore, the structural domains of all 12 nORFs and their potential homologs in related species were analyzed using online interPro tools and the BLASTP module. The domain analysis results indicated that four nORFs contained known domains, including signal peptides and transmembrane regions. Three nORFs were predicted to possess intrinsically disordered domains, while no defined structural features were identified in the remainder (Table 1). It is worth noting that both nORF8 and nORF9 contained a signal peptide sequence and a non-cytoplasmic domain (extracellular region), respectively, which suggested that they are putative secretory proteins (Fig. 3B). The coding regions of nORF8 and nORF9 were located within the predicted protein-coding sequence of ORF104, a conserved protein kinase. Besides, protein homology analysis suggested that the small protein nORF2 (15 amino acids) shared sequence similarities to the insect protein titin, which is involved in muscle movement (Fig. 3C). In conclusion, the above analysis supports the presence of the predicted nORFs in the CyHV-2 genome.

### Identification of phosphorylation modifications in CyHV-2 peptides

**Fig 4:**
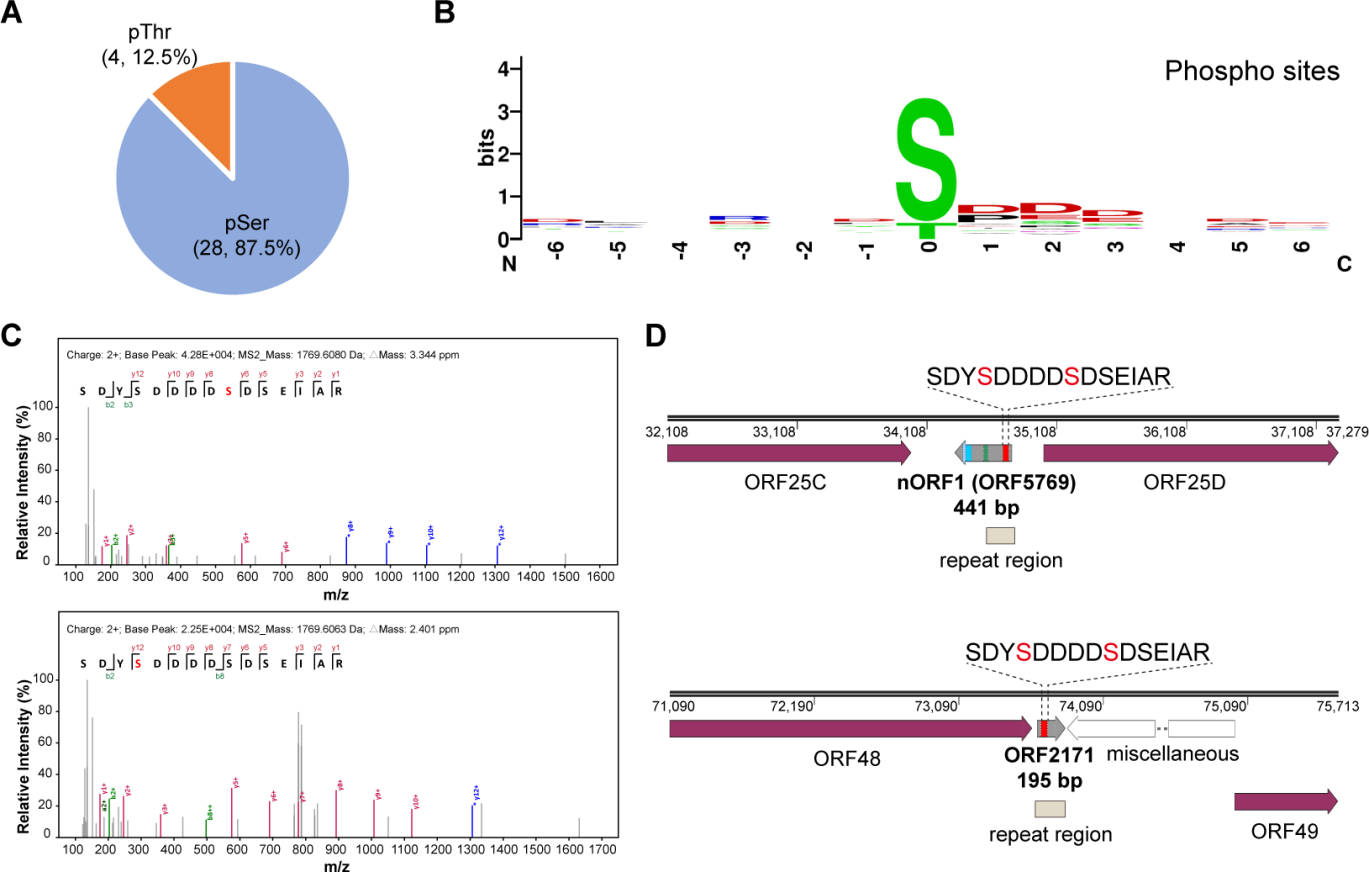
Phosphorylation analysis in CyHV-2. (**A**) Distribution of phosphorylation sites in CyHV-2. (**B**) Motif logos of 28 phosphorylated peptides identified in the kidneys of CyHV-2-infected crucian carp and captured using Ti^4+^-IMAC. (**C**) MS/MS spectra of the SDYSDDDDSDSEIAR peptide in nORFs possessing two phosphorylation sites. (**D**) Mapping of the SDYSDDDDSDSEIAR peptide in the CyHV-2 genome. The identified unique peptides of nORF1 are indicated in different colored boxes.

In our previous study, we conducted a phosphoproteomic analysis of CyHV-2-infected crucian carp. After enrichment by immobilized titanium ion affinity chromatography (Ti^4+^-IMAC), 11 viral proteins were identified to possess phosphorylation modifications (22). To more comprehensively assess the presence of phosphorylated sites in viral proteins, the primary MS/MS spectra were re-queried against the novel customized protein database using Open-pFind software. Finally, a total of 32 phosphorylated sites and 28 phosphorylated peptides were obtained, corresponding to 16 viral phosphorylated proteins, including 11 previously discovered and five novel CyHV-2 phosphorylated proteins (Table 2). Upon analyzing the identity of all phosphoamino acids, the proportions of pSer and pThr residues were found to be 87.5% and 12.5%, respectively, while no pTyr residues were identified, mirroring the findings in crucian carp (Fig. 4A). Sequence motif analysis revealed that the three primary successive amino acids downstream of pSer/pThr residues were aspartic acid, highlighting the important roles of aspartic acid for Ser/Thr phosphorylation in CyHV-2 (Fig. 4B).

**TABLE 2:** Modification sites in phosphorylated proteins and peptides in CyHV-2

Protein	Protein description	PSMs	Modification sites
nORF1	Membrane-bound protein	2	SDYSDDDDSDSEIAR
ORF31	PLAC8 family	1	MESIDVVR
ORF56	Protein ORF56	1	IAPQTTPPKTVEQDDPK
ORF121	Protein ORF121	1	SYSPEFNR
ORF125	Contains the transmembrane region and cytoplasmic domain	1	YSEDEDDR

The MS/MS spectra of all 28 phosphorylated peptides were manually checked and were accompanied by a comprehensive b/y ion series to support their reliability. A total of 27 of the 28 phosphorylated peptides were unique peptides that were mapped to a single CyHV-2 genome region. However, the peptide SDYSDDDDSDSEIAR possessing two validated pSer residues could map to two regions of the CyHV-2 genome that were downstream of ORF25C and ORF48 (Fig. 4C and D). The genomic location of this novel phosphorylated peptide was determined, as was its classification among CyHV-2 ORFs generated by the ORFfinder software. The sequence coverages of corresponding ORFs near ORF25C and ORF48 were analyzed as the detection of at least one unique peptide was necessary to support the existence of the predicted ORFs. We found that only one ORF in the downstream regions of ORF25C and ORF48 was predicted by ORFfinder software, which were ORF5769 and ORF2171, both containing the sequences of the novel phosphorylated peptide (Fig. 4D). Moreover, ORF5769 was renamed to nORF1 due to the identification of three distinct peptides by MS (Table 1), but no additional peptides could be matched to ORF2171 (Fig. 4D). Thus, it is reasonable to suggest that nORF1 undergoes phosphorylation modifications. All the viral phosphorylated proteins identified are presented in Fig. 2.

### nORF1 is a marker for the evolutionary divergence of CyHV-2

**Fig 5:**
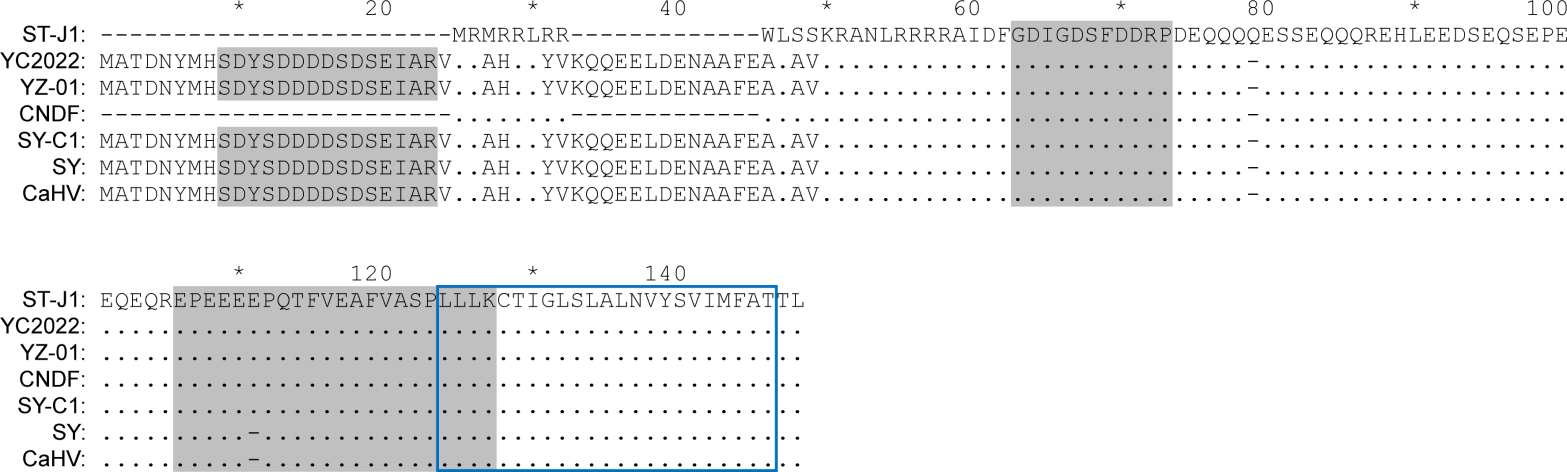
Multiple sequence alignment of nORF1 in different CyHV-2 strains. The sequences were obtained from the NCBI database and aligned using Clustal W. The identified peptides of nORF1 are highlighted in gray. The blue box indicates the predicted membrane-bound region.

An unusual 220 bp inverted repeat has been reported in the CyHV-2 ST-J1 genome, with copies downstream of ORF25C and ORF48 (1) (Fig. 4D). The repetitive sequence can also be found in specific genomic regions of the C genotype of CyHV-2, although the sequence exhibits variability to some extent. For example, downstream of ORF25C, four bases are deleted from the 220 bp repeat in CyHV-2 YZ-01, SY-C1, SY, and CaHV strains, yet there is an exact match between ST-J1 and CNDF strains [Fig. S3A]. A sequence insertion of a small fragment in a repetitive sequence was observed downstream of ORF48 in CyHV-2 SY-C1 and SY strains (Fig. S3B). Coincidentally, the coding region of nORF1 covers much of the 220 bp inverted repeat region, and the CDS of nORF1 was markedly altered in conjunction with the base deletion in the repetitive sequence (Fig. 4D). Multiple sequence alignment of the potential nORF1 in different CyHV-2 strains revealed that the N-terminal of nORF1 in CyHV-2 YZ-01, SY-C1, SY, CaHV, and YC2022 strains was significantly longer than in ST-J1 and CNDF strains (Fig. 5). The novel phosphorylated peptide was also found to be located in the N-terminus of nORF1, which was missing in ST-J1 and CNDF strains. Furthermore, the sequence of nORF1 in the CNDF strain showed a high similarity with that in the ST-J1 strain, which is an important feature in connecting the C genotype with the J genotype of CyHV-2 in the process of evolution.

**Fig 6:**
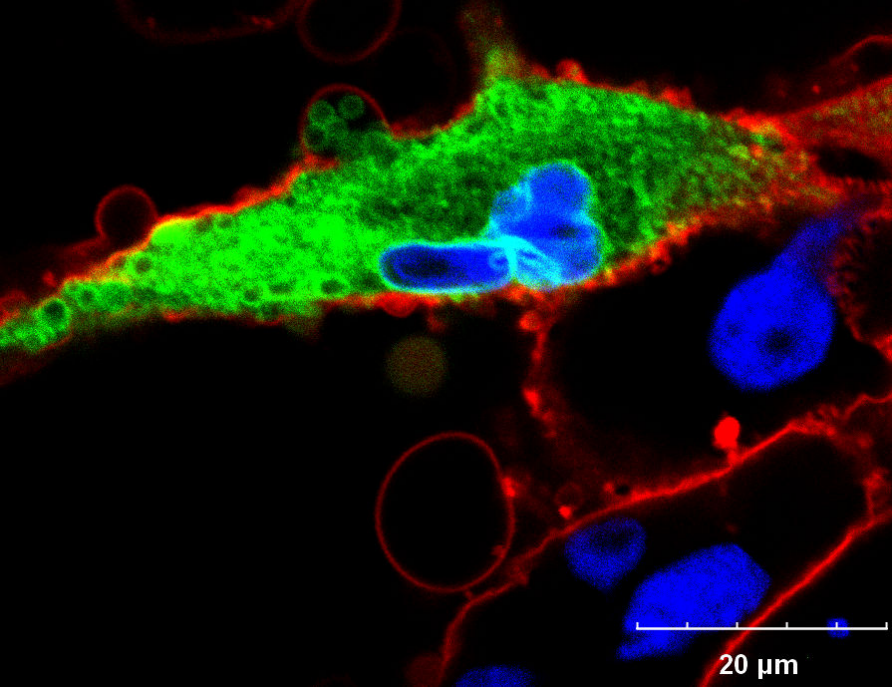
Subcellular localization of the nORF1-GFP in GiCB cells. The cell membrane and nucleus were stained with Dil and DAPI, respectively.

The membrane protein nORF1 belongs to the ORF25 family of CyHV-2, and no homologs have been found in other known herpesviruses. The ORF25 family, comprising ORF25, ORF25B, OEF25C, and ORF25D, is a group of membrane proteins involved in the interaction between CyHV-2 and the host (29). By expressing the recombinant nORF1-GFP in GiCB cells, we observed that the nORF1-GFP was closely associated with the cell membrane, could aggregate into a ring structure, and was mainly distributed in the nuclear membrane and cytoplasm. Accordingly, part of looped nORF1 appeared to have a tendency to fuse with the cell membrane and be released from within the cell (Fig. 6). Combining the genomic location and membrane-bound region of nORF1, we propose that the nORF1 can be classified into the ORF25 family and was renamed as ORF25E. The intact ORF25E is only found in some of the C genotypes of CyHV-2. Moreover, ORF25E is also absent from CyHV1, CyHV3, and other known herpesviruses. These results indicated that ORF25E may function as a significant evolutionary molecule between different CyHV2 isolates, leading to differences in the adsorption capacity on crucian carps.

## DISCUSSION

In this study, we performed an integrated proteogenomic analysis to amend the annotation of the CyHV-2 genome. We unambiguously identified 129 viral genes, including 117 currently annotated genes and 12 novel genes. For the first time, evidence of peptide presence and levels in the genome of aquatic viruses was presented on a large scale, and most of the predicted proteins in CyHV-2 were verified.

Several key features significantly enhanced the performance of our proteogenomic pipeline: (i) the application of two protein extraction buffers with different pH (50 mM HCl and Tris-HCl buffer) could complement each other to maximize the efficiency of protein identification as sORFs are more effectively enriched in HCl buffer; (ii) the reduction of sample complexity using high pH reverse-phase separation is beneficial to improve the identification efficiency of mass spectrometry, especially when following a bottom–up strategy; (iii) multiple database search engines were used for spectrum analysis against customized databases to increase peptide identification efficiency. However, certain factors that could not be adequately controlled persisted and impeded, to an extent, protein identification, such as host protein contamination, substitution of leucine and isoleucine, and single-amino acid variants. We conducted the proteogenomic analysis and CyHV-2 protein identification on the kidneys of CyHV-2-infected fish, rather than purified virus particles. Although viral proteins are preserved as much as possible in intact tissue, including structural and nonstructural proteins, high host protein levels inevitably dilute the viral protein abundance, which causes a reduction in the protein identification efficiency by MS. Besides, due to the equal MW of leucine and isoleucine, it is hard to obtain the exact sequence of the peptides that contain these two amino acids by MS. We have noticed that hundreds of peptides with a substitution of leucine with isoleucine could be mapped to the crucian carp or CyHV-2 proteome. This led to a decline in the number of viral proteins identified as these peptides of undetermined origin were eventually removed during the proteogenomic analysis. At the same time, neglecting single-amino acid variants between identified peptides and annotated peptides may also hinder viral protein identification. Thus, we believe that CyHV-2 might encode more proteins than are currently known.

Additional techniques need to be employed to enrich protein-level evidence of 27 unidentified ORFs in the current annotated genome, including the preparation of specific antibodies. On the one hand, antibodies can detect the target proteins directly by Western blot. On the other hand, immune-enriched target proteins can increase the identification efficacy of MS. For example, there is still a lack of peptide-level evidence for the conserved herpesvirus protein kinase (CHPK), CyHV-2 ORF104, but two novel ORFs were unexpectedly found in its protein-coding regions. Notably, CHPKs are a group of serine/threonine protein kinases that are encoded by all known herpesviruses and play a conserved role in viral infection by influencing the host’s protein synthesis, immune response, and the state of phosphorylation modifications (30, 31). Moreover, previous research has shown that CyHV-2 ORF104 possesses a nuclear localization signal, and after expression of its full-length gene, it was found to be localized in fish EPC cell nuclei (32). Thus, it is reasonable to suggest that the CyHV-2 ORF104 is expressed during viral replication. The preparation of antibodies for CyHV-2 ORF104 and other unidentified CyHV-2 proteins is an effective method to directly confirm their presence and contribute to their functional characterization.

The biological significance of CyHV-2 nORFs remains unclear. We searched the CDS of 12 nORFs against the NCBI database using the BLASTP module, but no obvious homologs were found in current species, suggesting that these nORFs may be specific to CyHV-2. Understanding the potential biological significance of nORFs is challenging without explicit protein structure information. Currently, we know that four nORFs (nORF1, nORF4, nORF8, and nORF9) contain distinct transmembrane regions or non-cytoplasmic domains. It is important to investigate whether these novel membrane proteins could serve as targets for antiviral drug development. For example, nORF1/ORF25E likely exhibits good immunogenicity, akin to other ORF25 family members, including ORF25 protein (13, 29, 33). And the GiCB cells blocked with ORF25 protein exhibit significant antiviral defense efficacy, although the identity of the host receptor that interacts with ORF25 remains unidentified (29). The function and immunogenicity of these novel membrane proteins need to be assessed in future studies.

Phosphorylation events influence viral replication and provide potential therapeutic targets. In antiviral therapy, in addition to developing highly effective neutralizing antibodies to bind and inactivate virions, inhibiting phosphorylation signaling pathways is also an effective approach to modulate viral replication (34, 35). Phosphorylation affects the structure and function of viral proteins, which are essential for optimal viral replication. Additionally, kinase inhibitors can reduce the negative effects of over-phosphorylation events stimulated by viral proteins (34). Many kinase inhibitors, including pharmacological inhibitors of CK2, p38 MAPK signaling, and CDKs, have been shown to possess strong antiviral efficacy (36, 37). In gibel carp, CyHV-2 infection enhances p38 phosphorylation, and the p38 inhibitor SB203580 significantly reduces fish mortality caused by CyHV-2 infection (32). In this study, a total of 32 phosphorylated sites corresponding to 16 viral proteins were identified, accounting for approximately 10% of the CyHV-2 genome. However, the responsible kinases remain unidentified. Determining the phenotypic effects of specific phosphorylations in CyHV-2 proteins using mutational analysis is crucial for identifying critical phosphorylation sites involved in viral replication. Moreover, the investigation of responsible kinases of CyHV-2 phosphorylated proteins will aid in selecting kinase inhibitors for prophylactic and therapeutic treatments of viral infections.

In conclusion, our proteogenomic data set highlights the value of incorporating experimental data into gene discovery and annotation. The discovery of novel genes serves as a valuable resource for elucidating the evolution of CyHVs and provides novel insights into the fields of pharmacology and vaccinology.

## Data Availability

The mass spectrometry proteomics data have been deposited in the ProteomeXchange Consortium (https://proteomecentral.proteomexchange.org) via the iProX partner repository with the dataset identifier PXD059229.

## SUPPLEMENTAL MATERIAL

Supplemental figures jvi.01960-24-s0001.docx Figures S1 to S3.

Table S1 jvi.01960-24-s0002.txt CyHV-2 ORFs translated in six reading frames.

Table S2 jvi.01960-24-s0003.docx RT-PCR primers for nORF amplification.

Table S3 jvi.01960-24-s0004.txt Sequences of eight highly variable genes in CyHV-2.

Table S4 jvi.01960-24-s0005.xlsx Identified peptides mapped to the CyHV-2 genome.

ASM does not own the copyrights to Supplemental Material that may be linked to, or accessed through, an article. The authors have granted ASM a non-exclusive, world-wide license to publish the Supplemental Material files. Please contact the corresponding author directly for reuse.
