# Supplementary material for: Proteogenomic analysis of Cyprinid herpesvirus 2 using high-resolution mass spectrometry
Source: J Virol. 2025 Apr 2;99(5):e01960-24. doi: 10.1128/jvi.01960-24 (PMC12090726; doi:10.1128/jvi.01960-24)
Supplement: Supplemental figures — Figures S1 to S3. [file jvi.01960-24-s0001.docx]

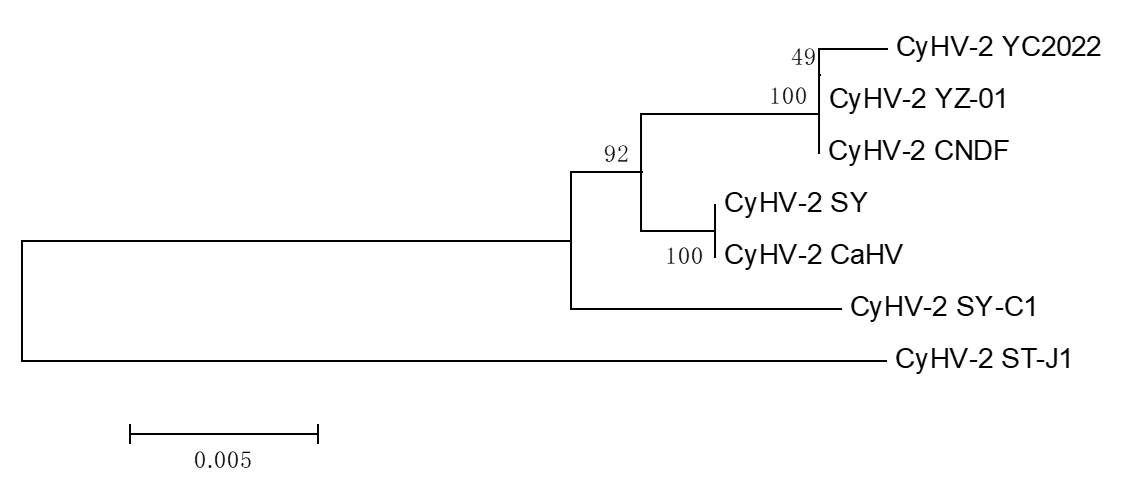


**Figure S1. Phylogenetic analyses of CyHV-2 YC2022 strain and other strains collected in NCBI database.** A neighbor-joining tree was constructed based on the sequences of eight variable genes (*ORF10*, *ORF25B*, *TK*, *ORF63*, *ORF71*, *ORF79*, *ORF107*, and *ORF156*), using MEGA 6.0 software with 1,000 bootstrap replications.


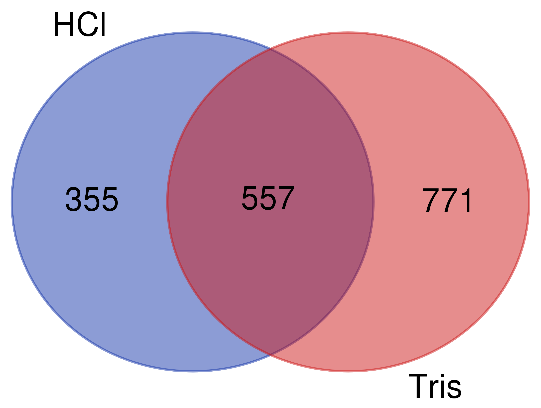


**Figure S2. The Venn diagram shows the peptide identification results obtained from the HCl and Tris extraction methods.**


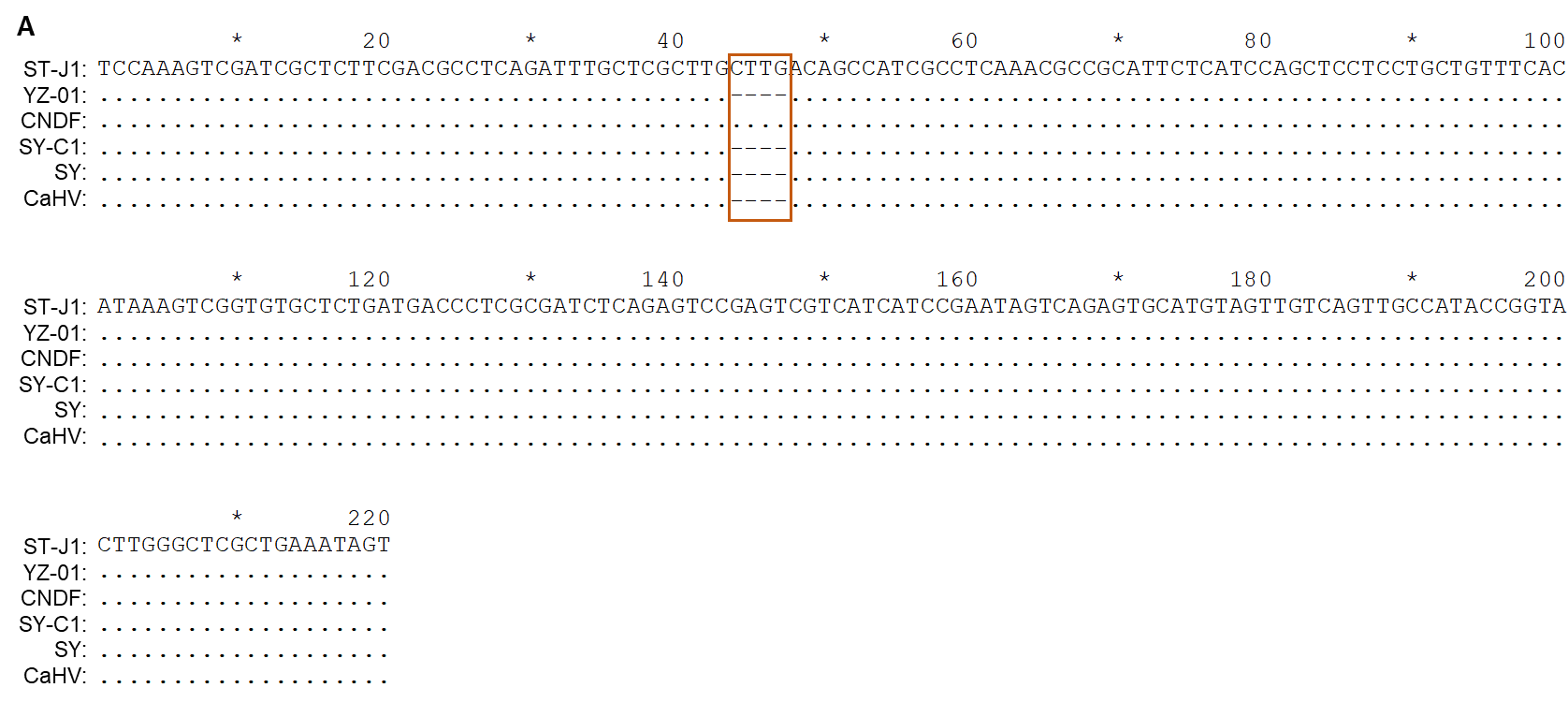


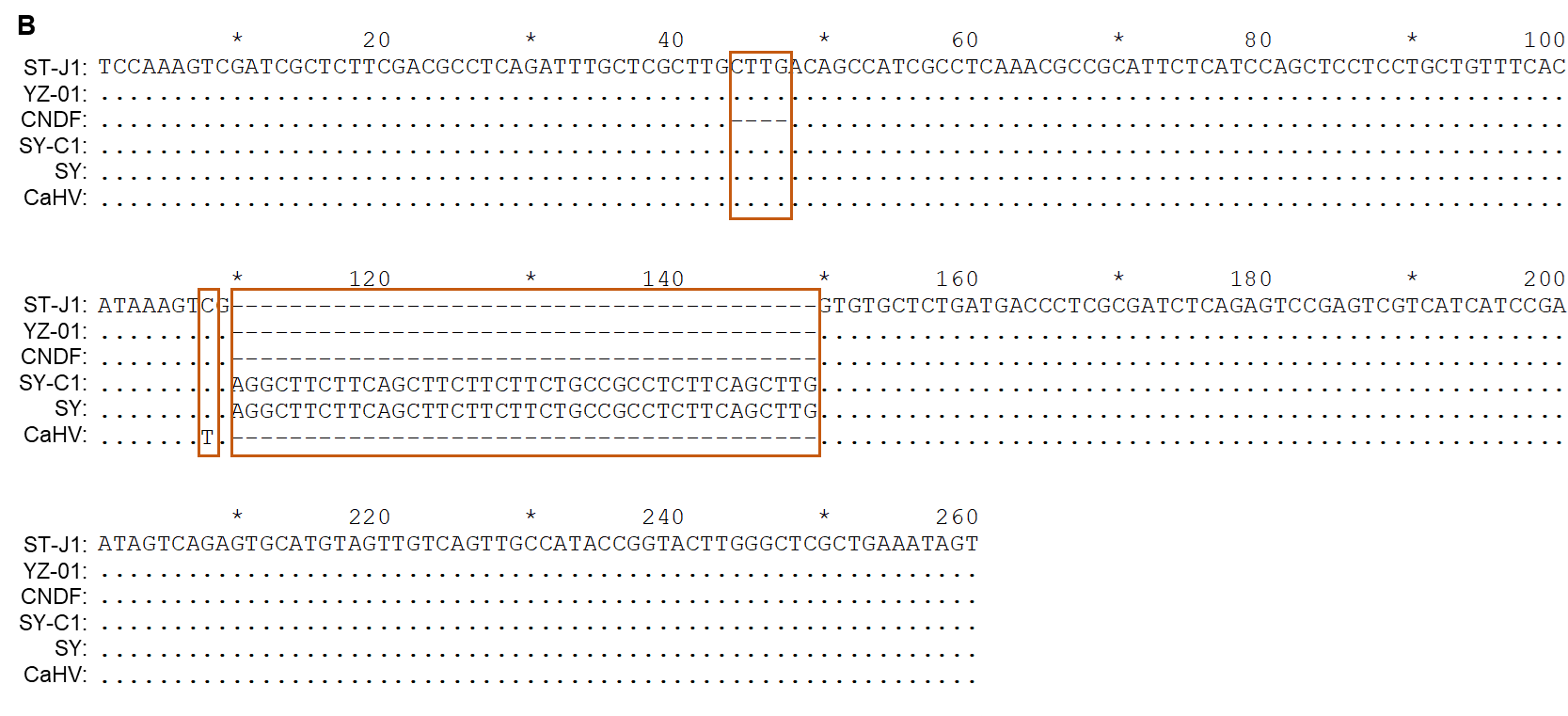


**Figure S3. Multiple sequence alignment of 220 bp inverted repeat in different CyHV-2 strains.** The repetitive sequence located on the right of ORF25C (A) and ORF48 (B), respectively. The differences include sequence deletion, insertion, and point mutation between different CyHV-2 strains were boxed in brown.
