# Supplementary material for: Proteogenomic analysis of Cyprinid herpesvirus 2 using high-resolution mass spectrometry
Source: J Virol. 2025 Apr 2;99(5):e01960-24. doi: 10.1128/jvi.01960-24 (PMC12090726; doi:10.1128/jvi.01960-24)
Supplement: Table S2 — RT-PCR primers for nORF amplification. [file jvi.01960-24-s0003.docx]

**TableS2. Gene-specific primers used for RT-PCR**

| **Gene** | **Primer Sequence (5’-3’)** | |
| --- | --- | --- |
|  | **Forward primer** | **Reverse primer** |
| *nORF1* | ATGGCAACTGACAACTACATG | TAAAGTAGTAGCAAACATAATCAC |
| *nORF2* | AGAATCTCAAGGGAGTCCG | ACCAGGTGGTTTCCAGTTT |
| *nORF3* | AGGACTTGGTTATCAGCACAACAG | AGTCCTCTTGTGCTGCTGTTG |
| *nORF4* | CTCACTTTGACGATTGTTGTAAC | CTTTAGCGTGATGCCAGCCTG |
| *nORF5* | ATGCCTGAACAACCAGCAGCAG | GCACGCTTATCACGATGTCCTTG |
| *nORF6* | ATGCCATCTCTGGAGGTACTAC | AAACCCCGGCTCCCGCGCCTC |
| *nORF7* | ATCTTCTCTGAGGTGATCCGGG | ACCATGAAAAAATTGACGTGTC |
| *nORF8* | TTTAGAGTACGCTTGATGGTATC | AGTAGTCGATCTGATCTCGCC |
| *nORF9* | AGTACTCCCAGTATAATCTCGC | TTCGGACGATGACAAGCCTAC |
| *nORF10* | GAAGCTGACCCTCTCCGGACTC | TCGTTCTGATTTTTTTTTATGTTG |
| *nORF11* | ATGTGGACGATCACAAACACAAG | AACCACGAGGCATAAAGACATCC |
| *nORF12* | ATGATTCTAGCTCTAGTTCAAG | CTGTGTTGGGTGCAGGGGACG |
